# Effects of Different Laying Hen Species on Odour Emissions

**DOI:** 10.3390/ani10112172

**Published:** 2020-11-21

**Authors:** Dongdong Lu, Jiandui Mi, Yinbao Wu, Juanboo Liang, Xindi Liao, Yan Wang

**Affiliations:** 1 College of Animal Science, South China Agricultural University, Guangzhou 510642, China; dongd.lu@hotmail.com (D.L.); mijiandui@163.com (J.M.); wuyinbao@scau.edu.cn (Y.W.); 2 Guangdong Provincial Key Lab of Agro-Animal Genomics and Molecular Breeding, South China Agricultural University, Guangzhou 510642, China; 3 Institute of Tropical Agriculture and Food Security, Universiti Putra Malaysia, Serdang 43400, Malaysia; jbliang@upm.edu.my

**Keywords:** ammonia, hydrogen sulphide, laying hen, species, in vitro fermentation

## Abstract

**Simple Summary:**

Odour emissions from the laying hen industry receive considerable attention because they influence the air quality, the efficiency of animal production and the health of workers. Recently, numerous researchers have hypothesized that the choice of animals may influence the emission of gases, which may be utilized as the basis for gas pollution reduction strategies in the future. The present study employed an in vitro fermentation technique to simulate gas production from the caecum to explore the effects of laying hen species on the production of ammonia (NH_3_) and hydrogen sulphide (H_2_S). The results showed that the Xinghua laying hen had the lowest odour gas production among the six laying hen species tested. Spearman correlation analysis showed that odour production was associated with enzyme activities but was not significantly associated with specific enzyme genes. The results of this study provide useful information for odour reduction in the laying hen industry.

**Abstract:**

Odour is one of the main environmental concerns in the laying hen industry and may also influence animal health and production performance. Previous studies showed that odours from the laying hen body are primarily produced from the microbial fermentation (breakdown) of organic materials in the caecum, and different laying hen species may have different odour production potentials. This study was conducted to evaluate the emissions of two primary odorous gases, ammonia (NH_3_) and hydrogen sulphide (H_2_S), from six different laying hen species (Hyline, Lohmann, Nongda, Jingfen, Xinghua and Zhusi). An in vitro fermentation technique was adopted in this study, which has been reported to be an appropriate method for simulating gas production from the microbial fermentation of organic materials in the caecum. The results of this study show that Jingfen produced the greatest volume of gas after 12 h of fermentation (*p* < 0.05). Hyline had the highest, while Lohmann had the lowest, total NH_3_ emissions (*p* < 0.05). The total H_2_S emissions of Zhusi and Hyline were higher than those of Lohmann, Jingfen and Xinghua (*p* < 0.05), while Xinghua exhibited the lowest total H_2_S emissions (*p* < 0.05). Of the six laying hen species, Xinghua was identified as the best species because it produced the lowest total amount of NH_3_ + H_2_S (39.94 µg). The results for the biochemical indicators showed that the concentration of volatile fatty acids (VFAs) from Zhusi was higher than that for the other five species, while the pH in Zhusi was lower (*p* < 0.01), and the concentrations of ammonium nitrogen (NH^4+^), uric acid and urea in Xinghua were lower than those in the other species (*p* < 0.01). Hyline had the highest change in SO_4_^2−^ concentration during the fermentation processes (*p* < 0.05). In addition, the results of the correlation analysis suggested that NH_3_ emission is positively related to urease activities but is not significantly related to the ureC gene number. Furthermore, H_2_S emission was observed to be significantly related to the reduction of SO_4_^2−^ but showed no connection with the aprA gene number. Overall, our findings provide a reference for future feeding programmes attempting to reduce odour pollution in the laying hen industry.

## 1. Introduction

The poultry industry is an important economic industry in many countries. It has been reported that the number of laying hens in the world increased from 4.973 billion to 7.891 billion, representing an increase of 58.67%, from 2000 to 2018, and at the same time, the number of laying hens in China increased from 1.931 billion to 3.12 billion, representing an increase of 61.58% [1]. Recently, with the increase in poultry population, odour pollution in the laying hen industry has been a source of increasing concern from the general public because this pollution has the potential to affect regional air quality [2]. Studies have shown that odours are harmful for the air quality in animal feeding operations (AFOs) [3,4], which also influences the well-being and production performance of the animals [5,6]. Moreover, the extended exposure of the workers to these contaminants is associated with an increased risk of respiratory diseases [7].

Ammonia (NH_3_) and hydrogen sulphide (H_2_S) are the two main odour polluters in the laying hen industry, and they are the key targets in the research of poultry gas pollution reduction. NH_3_ is an invisible, water-soluble alkaline gas that is recognized as one of the most prominent contaminants, and a concentration of NH_3_ greater than 25 ppm can stimulate the mucosa and respiratory tracts of birds, causing respiratory disease and heat disease [8,9]. Additionally, NH_3_ contributes to greenhouse gas emissions, since NH_3_ is a precursor of nitrous oxide [10]. H_2_S, which has a characteristic ‘‘rotten egg’’ smell, is one of the predominant noxious gases and is second only to NH_3_ in animal production because of its low odour threshold and high toxicity [11,12]. The exposure of animals to certain concentrations of H_2_S has been linked to liver, spleen and respiratory diseases and immune system damage [13,14,15]. Exposure to more than 20 ppm of H_2_S may lead to activated inflammatory responses and higher relative weight losses of the bursa, spleen and thymus in chickens [16].

The fermentation of organic compounds by caecal microorganisms is the main source of body odour in laying hens [17,18]. More specifically, fermentation in the gut can be divided into two types: carbohydrate fermentation and nitrogen-containing fermentation [4]. The metabolites of the different fermentation types are different and are directly related to odour emission. For example, NH_3_ is a by-product of the microbial decomposition of organic nitrogen compounds, and part of the NH_3_ is derived from the deamination of amino acids by microorganisms, but most of it is derived from the decomposition of urea by microbial urease [8]. H_2_S mainly results from the fermentation of undigested sulphur protein in the hindgut, as well as the reduction of oxidized inorganic sulphur compounds, such as sulphate radicals (SO_4_^2−^), by sulphur-reducing bacteria (SRB) [17,19]. There are many bacterial genera involved in sulphate reduction or protein degradation, such as *Desulfovibrio*, *Veillonella*, *Megasphaera* and *Enterobacteria* [20,21,22]. A previous study indicated that, under similar environments, host species have the potential to affect the composition of intestinal microorganisms, including some gas-producing ones [23,24]. Additionally, Rahman found that species may affect N loss when comparing the N retention of Hyline and Lohmann hens [25]. Therefore, it is hypothesized that different kinds of laying hen have different odour emission potentials.

To test this hypothesis, six laying hen species were selected, based on the practices and classification of the current laying hen industry in China for this study. These six species were Lohmann and Hyline (imported commercial species), Nongda and Jingfen (hybrid commercial species), and Zhusi and Xinghua (Chinese indigenous species), and all of them are widely farmed in China. In this study, the odour gas emissions from the six laying hen species were measured using an in vitro fermentation technique that has been reported as a reliable procedure for simulating gas production resulting from microbial fermentation in the caecum. In addition, the underlying mechanisms of the differences between different species were elucidated in this study. To the best of our knowledge, this study is the first to compare odour gas emissions from different species of laying hen, which could provide a reference for future breeding or selection programmes attempting to reduce gas emissions in the laying hen industry.

## 2. Materials and Methods

### 2.1. Ethics Statement

This experiment followed the institutional guidelines for the care and use of animals, and all experimental procedures involving animals were approved by the Animal Experimental Committee of South China Agricultural University (Ethics Approval Code: SYXK 2014-0136).

### 2.2. Animal Management and Preparation of Inoculums

A total of 120 laying hens of six different species (20 birds/species), 40 weeks old, from the same farm (Wens Nanfang Poultry Breeding Co. Ltd., Yun Fu, China) were used for this study. On arrival at the experimental farm, hens within each species were randomly allocated in equal numbers (4 hens/cage) with 5 cages/replicates (*n* = 5 hens per species). The laying hens in all the cages were given free-choice access to a standard corn–soybean-based diet formulated to meet the nutrient requirements of the National Research Council (NRC) [26], and the diet is shown in Supplementary Materials, Table S1. All birds were provided with clean drinking water and identical experimental conditions. The indoor temperature was maintained at 24 °C, and the average humidity was 79.1% throughout the experiments. In the feeding stage, production performance indexes, such as the egg yield, egg weight, average daily feed intake (ADFI), body weight (BW) and number of broken eggs, were recorded daily on a cage basis, and the feed conversion ratio (FCR) was calculated.

**Figure 1:**
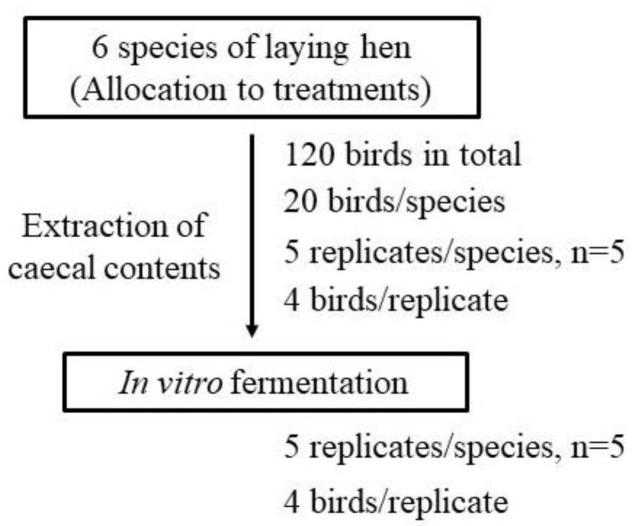
The experimental design, showing treatments and replicates.

All the hens were slaughtered after 28 days in the feeding trial. The caecal contents of 4 laying hens from the same cage were collected and pooled into one inoculum (replicate) to provide a total of 5 replicates per treatment (species). The pooling of the caecal content from 4 hens within each replicate was to ensure that sufficient sample material per replicate was collected for the fermentation study, and this is practiced in many microbiology studies [27]. The experimental procedure, showing the 6 treatments (species) and 5 replicates/treatment, is shown in Figure 1. Each pooled inoculum was individually mixed thoroughly with sodium and ammonium bicarbonate buffer solution (35 g of NaHCO_3_ plus 4 g of NH_4_HCO_3_ per L) in a 1:3 (*w*/*v*) ratio. The intestinal and buffer mixtures were individually squeezed through four layers of surgical gauze into a bottle and were continuously bubbled with CO_2_ at 39 °C.

### 2.3. In Vitro Fermentation and Sample Collection

The in vitro fermentation technique employed in this study has been reported to be a reliable technique for simulating gas production from the intestinal microbial activity of laying hens [18]. Briefly, the technique was based on the in vitro gas production procedure described by Menke and Steingass [28], with adaptations for use with chickens following Wang et al. [18]. Each treatment group was replicated with five syringes and one control. Each syringe was used as an experimental unit. The fermentation inoculum was prepared accordingly, and 30 mL of it was added to a 100 mL glass syringe (Häberle, Schwerte, Germany) containing 500 mg of the experimental diet, while the control was prepared by only adding the inoculum. After removing the air from the headspace, the syringes were sealed with clamps, placed in an incubator at 39 °C and rotated at 60 rpm for 12 h. This experiment was performed in two runs. After 12 h, the fermentation was terminated by transferring the syringes into an ice box, and the final reading of each syringe was recorded. The gas accumulated in the headspace of each syringe was collected with a gastight syringe (Hamilton, Reno, NV, USA) and immediately transferred into a Teflon gas bag used for the determination of NH_3_ and H_2_S emissions. The fermentation solution was transferred to a 50 mL centrifuge tube and centrifuged at 200× *g* at 4 °C for 5 min. The supernatant was separated, transferred into another 50 mL centrifuge tube and stored at −80 °C for further analysis.

### 2.4. Odour Gas Measurements

Ten millilitres of gas was extracted from the gas bag and then slowly injected into a sulphuric acid solution containing cadmium sulphate solution to ensure adequate absorption. The NH_3_ collected in the sulphuric acid solution was determined with a spectrophotometer (Shanghai Aoyi Technology Co., Ltd., Shanghai, China) based on the Chinese National Environmental Protection Standards (determination of ammonium nitrogen—Nessler’s reagent spectrophotometry). The containing cadmium sulphate solution was immediately added to 1 mL of mixed developer for the determination of hydrogen sulphide based on the Chinese National Environmental Protection Standards (determination of hydrogen sulphide in air—methylene blue spectrophotometric method).

### 2.5. Analysis of Fermentation Liquid Samples

The turbidimetric method was used to determine the concentration of SO_4_^2−^ in the inoculum before and after fermentation [29]. The urease activity was determined using colorimetry according to Guan [30]. The uric acid and urea in the supernatant were determined using a detection kit (Nanjing Jiancheng Bioengineering Institute, Nanjing, China) according to the instructions. The pH value was measured using a digital pH meter with a 1.5 mm microelectrode, and the concentration of volatile fatty acids (VFAs) was determined using a gas chromatograph (GC-2010; Shimadzu, Kyoto, Japan) equipped with a flame ionization detector.

### 2.6. DNA Extraction and aprA and ureC Gene Quantification

To determine the effect of species on the function of the microbial genes, the numbers of urease-producing bacteria and sulphate-reducing bacteria in the post-fermentation solution were quantified. The total DNA from each sample was extracted using an EZNATM Stool DNA Kit (Omega Bio-Tek Inc., Norcross, GA, USA). The integrity of the DNA samples was examined by electrophoresis on a 1% agarose gel. Then, the DNA purity was determined by measuring the absorbance ratio of a sample at 260 and 280 nm using an ultrafine ultraviolet (UV) spectrophotometer (for the samples, the OD260/OD280 ratio of the DNA was required to be between 1.8 and 2.0). The marker gene aprA (adenosine-5′-phosphosulphate reductase alpha subunit gene) of the SRB and the functional gene ureC (urease C) of the urease-producing bacteria were quantified using real-time quantitative (q)-polymerase chain reaction (RT-PCR). The extracted DNA was used as a PCR template for real-time quantification, performed on an ABI 7500 instrument (Thermo Fisher Scientific Inc., Waltham, MA, USA). The primers for the two genes were synthesized by Shanghai Shenggong Bio Co., Ltd. (Shanghai, China), as follows: aprA, 5′-TGGCAGATMATGATYMACGG-3′ (forward) and 5′-GGGCCGTAACCGTCCTTGAA-3′ (reverse); ureC, 5′-GCATGCAATTGAATAAAGCC-3′ (forward) and 5′-GCCGCTATAACGGATCAAAT-3′ (reverse). Specific operation procedures were employed as described by Deng et al. [29]. The results are expressed as logarithmic values (log copies/mL) of gene copies per millilitre of fermentation liquid.

### 2.7. Statistical Analysis

Each fermentation syringe was defined as a replicate, and each species had 5 replicates. The data are expressed as the means with standard errors of the mean (SEM) and were analysed with one-way analysis of variance (ANOVA) using general linear model procedures of the SAS (SAS Institute Inc., Cary, NC, USA) statistical software package. Duncan’s multiple comparisons were conducted when significant differences were observed. For all the tests, differences were considered significant when *p*-values < 0.05 were obtained. In addition, the correlations of the odour emission variables with biochemical indicators of the fermentation broth were analysed by the Spearman correlation method in SAS.

## 3. Results

### 3.1. Odour Gas Production

The results for odour production are shown in Table 1. Overall, NH_3_ production was 2–6 times that of H_2_S production in the caecum of the laying hens, and Jingfen produced the highest gas volume; Lohmann showed the lowest gas volume production (*p* < 0.05). In terms of NH_3_ production, the concentration of NH_3_ produced by Hyline was the highest (*p* < 0.05). This result is consistent with the total NH_3_ results. Hyline represents the highest total NH_3_ but shows no significant difference from Nongda and Zhusi. Xinghua has the lowest total NH_3_ production. For H_2_S production, Zhusi is the species that produces the highest total H_2_S, while Lohman produces the lowest H_2_S. Interestingly, Hyline produced the highest concentration of H_2_S, while Xinghua produced the lowest concentration of H_2_S. Moreover, Hyline exhibited the largest total NH_3_ + H_2_S production (92.45 µg), while Xinghua produced the least (39.94 µg), after 12 h of fermentation (*p* < 0.05).

### 3.2. Production Performance

The production performance data are presented in Table 2. After 28 days of the feeding trial, the ADFIs of Hyline, Lohmann and Jingfen were higher than those of Xinghua and Zhusi (*p* < 0.05) but showed no differences compared with one another. Lohmann had the largest ADFI, and Xinghua had the lowest ADFI, among these species. In terms of egg production, Hyline had the highest rate of egg production, approximately 97.22%, while there were no significant differences compared with Lohmann (*p* > 0.05). Nongda had the lowest egg production rate (83.96%). The egg weight of Hyline was similar to that of Lohmann. Although the egg weight of Nongda was lower than that of Hyline, Lohmann and Jingfen, it was still higher than that of Xinghua and Zhusi. Moreover, Hyline had the lowest FCR, meaning that it could use feed more efficiently than the other five species, whereas Zhusi had the highest FCR. Overall, the ADFI, egg weight and egg production in the imported commercial species were higher than those in the hybrid commercial species and Chinese indigenous species. The FCR in the imported commercial species was lower than that in the hybrid commercial species and Chinese indigenous species, except for that of Lohmann, which was higher than that in Jingfen.

### 3.3. VFA Production in the Laying Hen Caecum

The VFA production in the fermentation liquid is shown in Table 3. Zhusi produced more total VFAs than Lohmann, Nongda, Jingfen and Xinghua (*p* < 0.05) but showed no difference from Hyline. Moreover, Zhusi produced the highest amounts of all kinds of VFAs and total VFAs, while it showed no difference from Hyline in propionic acid, isobutyric acid, isovaleric acid and valeric acid. In addition, Hyline exhibited the second-highest amount of VFA production after Zhusi. Xinghua exhibited the lowest production of all kinds of VFAs and total VFAs (*p* < 0.05).

### 3.4. Biochemical Indexes in Fermentation Liquid among Different Species

This study also investigated the nitrogen metabolism activity because that may be related to NH_3_ production in the caecum (all the results are shown in Table 4). The results show that Xinghua had the highest pH value (*p* < 0.05), which was approximately 0.3 units higher than that of Lohmann. There were no significant differences between Hyline and Lohmann in pH (*p* > 0.05). The amounts of ammonium nitrogen and uric acid in the caecum are presented in Table 4. Xinghua had the lowest ammonium nitrogen concentration (0.28 mg/mL). No difference was found between Hyline, Lohmann, Jingfen, Xinghua and Zhusi. Urease activity in Lohmann and Hyline was higher than that in the other four species, Nongda, Jingfen, Xinghua and Zhusi (*p* < 0.05). There were no significant differences in urease activity between Nongda, Jingfen, Xinghua and Zhusi (*p* > 0.05). The uric acid levels in Hyline, Lohmann, Nongda and Jingfen were higher than those in Xinghua and Zhusi. Regarding the caecal urea concentration, Xinghua had the lowest urea concentration in the caecum. There were no significant differences among Hyline, Lohmann and Nongda (*p* > 0.05).

### 3.5. Changes in Sulphate (SO_4_^2−^) Concentration in the Caecum

**Figure 2:**
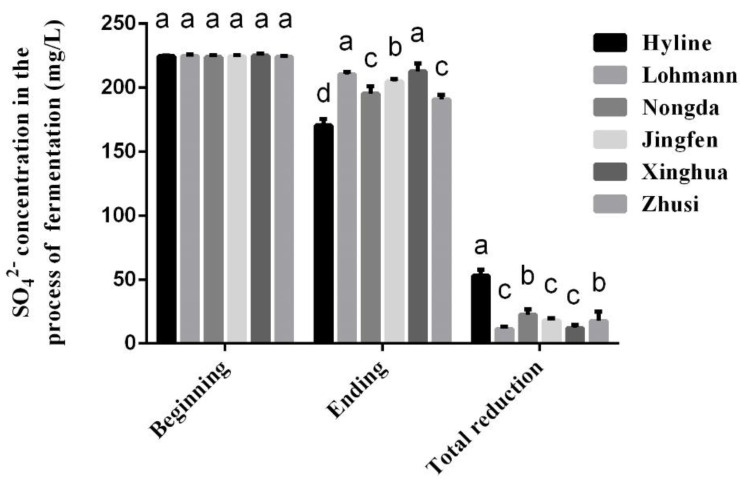
The changes in SO_4_^2−^ concentration during the fermentation processes (*n* = 5). Superscript letters a-d indicate differences between means within each species (*p* < 0.05).

The changes in the SO_4_^2-^ concentration during the fermentation processes are presented in Figure 2. All the species were calibrated at the same level of 225 mg/L at the beginning of fermentation. After 12 h of fermentation, the SO_4_^2−^ concentration of Hyline at the end was significantly lower than that of the other five species. This change is also shown as the result of the total SO_4_^2−^ concentration reduction. The reduction of SO_4_^2−^ in Hyline was the highest, followed by Nongda and Zhusi. In addition, SO_4_^2−^ in Lohmann was reduced the least, and there were no significant differences compared with Jingfen and Xinghua (*p* > 0.05).

### 3.6. Quantitation of the Gene UreC and Gene AprA

**Figure 3:**
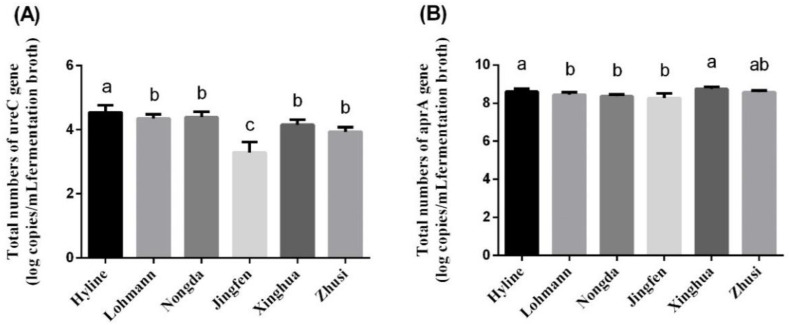
The quantitation of the gene ureC and gene aprA in six laying hen species (*n* = 5). (**A**) Total number of ureC genes. (**B**) Total number of aprA genes. Superscript letters a-c indicate differences between means within each species (*p* < 0.05).

The quantitative results for the functional gene ureC and marker gene aprA are presented in Figure 3. The number of ureC in Hyline was significantly higher than that in the other five species, with no difference being observed between Lohmann, Nongda, Xinghua and Zhusi (*p* > 0.05), whereas the number of ureC genes in Jingfen was the lowest (*p* < 0.05). According to the aprA gene quantitation, the number in Hyline was higher than that in Lohmann, Nongda and Zhusi (*p* < 0.05) but was not significantly different from that in Jingfen and Xinghua (*p* > 0.05). In addition, there was no significant difference in the number of aprA genes in Lohmann, Nongda and Zhusi (*p* > 0.05).

### 3.7. Correlation of Odour Emission with Intestinal Activities, Microbial Genes and Production Performance

The results of the analysis of the correlation between odour production and intestinal activities within these species are shown in Table 5, and the correlation with production performance is shown in Supplementary Materials, Table S2. The Spearman’s coefficient between the total volume and urease activity was −0.943, which means that the total gas production volume was strongly negatively correlated with urease activity and uric acid (*p* < 0.05). The Spearman’s coefficients between the NH_3_ concentration and ammonium nitrogen, total NH_3_ and uric acid were 0.771 and 0.771, respectively. This finding means that there are positive correlations among these indexes (*p* < 0.1). Additionally, the reduction in SO_4_^2−^ was significantly positively correlated with the concentration of H_2_S and total H_2_S (the Spearman’s coefficient was 0.812, *p* < 0.05). In addition, there was no correlation between odour emission and laying hen production performance (*p* > 0.05).

## 4. Discussion

At present, odour emissions from livestock farms are attracting increasing attention because of their negative impact on the surrounding environment and animal welfare. Some researchers recently reported that species of hens affect odour production in the laying hen industry and that gas emissions may be controlled by species selection [31]. However, little is known about the differences in odour production among different laying hen species, and the mechanisms governing these differences have not been elucidated. The primary objective of this study was to ascertain whether species affect odour emissions from laying hens and which species emits the lowest amounts of odorous gases. It is anticipated that the results of this study will provide a reference for future selection programmes for reducing odour gas emissions from the laying hen industry.

In the present study, the results show that species can affect odour production in laying hens. Overall, Zhusi and Hyline produced the highest total NH_3_ and total H_2_S, respectively, while Xinghua and Lohmann produced the lowest NH_3_ and H_2_S emissions, respectively. According to the total odour data (NH_3_ + H_2_S production), Xinghua was the best laying hen species (39.94 µg), while Hyline produced the largest amount of odorous gases (92.45 µg). The differences in NH_3_ production were also indicated by the concentration of ammonium nitrogen in the fermentation fluid because ammonia nitrogen is consistent with ammonia emissions in animal production [32]. Interestingly, Jingfen produced the highest total gas volume (39.11 µg), whereas it had low total odorous gas (85.43 µg). These results indicate that producing a higher volume of gas does not mean emitting more odour, which may be observed because the total gas not only includes odorous gases but also contains some odourless gases, such as methane, carbon dioxide and nitrous oxide [33,34].

Nitrogen and sulphur metabolism may be related to odour production in different species. Rahman found that Hyline had more NH_3_-N loss than any of the Lohmann brown strains [25]. Additionally, different types of laying hen species may produce different odours because of their differences in digestion. In this study, Xinghua and Zhusi are egg- and meat-type species that have higher NH_3_ emissions than commercial Lohmann. Steenfeldt and Hammershøj [35] compared the nitrogen metabolism between the commercial egg-type species Lohmann and egg- and meat-type species New Hampshire and found that the N content in laying hen excreta was significantly influenced by species; more specifically, Lohmann had a lower N excretion but a higher N retention than the egg- and meat-type species. At present, there is no research about the effect of species on the H_2_S emissions of laying hens, but sulphur emission is reported to be consistent with nitrogen emission [36,37]. These correlations were also confirmed in our study, according to the Spearman’s correlation between H_2_S and NH_3_ (Supplementary Materials, Table S2).

To better explain the role of caecal activities in digestion and odour production, the fermentation of the caecum needs further research. Odours released from animals have previously been linked to the enteric fermentation of feed protein; therefore, odour emission is also related to the retention of some organic substances in the hindgut [38,39]. The odours in laying hens are mostly from the hindgut, especially the caecum, where many gas-producing microorganisms colonize [40]. Therefore, intestinal odour production mainly depends on the biological reaction of microorganisms and microbial enzymes. Based on the theories outlined above, intestinal microbial activities may be the main cause of odour differences between the different species. The correlation analysis results in this study showed that both NH_3_ and H_2_S were positively correlated with total VFAs (r = 0.771, *p* < 0.1). Undigested protein fermentation in the caecum is a major source of odours, and the fermentation of these kinds of protein may produce potentially odorous end products, including indoles, NH_3_, H_2_S and branched-chain fatty acids [41]. Additionally, protein fermentation is often reported to be accompanied by carbohydrate fermentation. Zhusi produced the highest H_2_S and second-highest NH_3_, also producing the highest branched-chain VFAs, in this experiment. Branched VFAs, such as valeric acid, are metabolites of polypeptide fermentation after deamination, which has a strong correlation with odour production [42,43]. The intestinal pH value, which is connected to VFA production, is affected by the activity of some organic-acid-producing microbes, such as *Lactobacillus* and *Bacillus* [44,45]. Interestingly, no correlation between VFAs and the pH value was found, and this phenomenon may also be attributable to alkali-producing microorganisms, such as *Sutterella* [46]. Additionally, the correlation index indicates that total NH_3_ emission is negatively related to the pH value. This phenomenon may result from NH_3_ trapped in the liquid as ammonium ions and from the way it takes up hydrogen from the liquid, which causes a higher pH value.

Intestinal fermentation is mainly due to the function of microorganisms. Previous studies indicated that host species can affect the composition of intestinal microorganisms, including some gas-producing microorganisms, under the condition that all environmental factors are held consistent [23,24]. The caecal microbiomes of indigenous Indian Aseel and Kadaknath chicken species were compared with those of the global commercial broiler Cobb400 and Ross 308 lines; the genera *Campylobacter*, *Lactobacillus* and *Bacillus* and *H. pylori,* which are known as potential gas-producing microorganisms, markedly differed between the indigenous Indian chicken species and global commercial species [47]. In this study, some biological parameters of the caecum connected to microbial activities, such as pH, urease activity and VFAs, were also affected by the species. To further understand the microbial activities, some genes related to microbial enzyme production were quantified in this study. Urease can catalyse the decomposition of urea into NH_3_ and carbonic acid, which is considered one of the NH_3_ production pathways [48]. The urease genes of microorganisms were mostly from Proteobacteria, such as *H. pylori* and *Campylobacter*, which produce urease [49,50]. The gene ureC is widely used to study NH_3_ emissions in agriculture as the main urease marker gene [48]; this was measured by RT-qPCR, and the results are shown in Figure 3a. Interestingly, the correlation analysis results show that there was no significant correlation between ureC and NH_3_ production. This finding may be observed because the activities of other ammonia-producing pathways, such as the deamination or transamination of amino acids, such as glutamine metabolism, are higher than urease production [51,52,53]. Different species have different abilities to reduce sulphate (SO_4_^2−^). Some SRBs, such as *D. vulgaris*, can use SO_4_^2−^ as the principal terminal electron acceptor, thereby generating sulphide in a process designated dissimilatory sulphate reduction [54], which can also ferment cysteine and methionine to produce sulphur-containing malodorous molecules, such as H_2_S and CH_3_SH [29]. Therefore, the functional gene aprA was quantified by RT-PCR because it encodes key enzymes (adenosine 5′-phosphosulphate reductase, APR) of dissimilatory sulphate reduction, appropriate for determining the number of SRB in the gut [55]. Interestingly, the correlation analysis showed that H_2_S is positively correlated with the reduction of SO_4_^2−^ but has no significant relationship with the quantitative number of aprA, which may be observed because there are some other enzymes in the caecum that have the function of reducing sulphate other than APR, such as adenosine 5′-phosphosulphate kinase and γ-glutamylcysteine ligase [56]. However, this hypothesis requires further verification.

## 5. Conclusions

To the best of our knowledge, this study is the first to determine differences in odour gas production among different laying hen species. The results show that Xinghua produced the lowest odour among the six laying hen species, while Hyline produced the highest odour. The results of this study offer a reference for future species selection programmes attempting to reduce gas emissions and improve the balance between laying hen production and the environment.

## Figures and Tables

**Table 1:** *In vitro* fermentation odour production in six species of laying hens (*n* = 5).

Species	Total Volume (mL)	Concentration of NH_3_ (µg/L)	Concentration of H_2_S (µg/L)	Total NH_3_ Emission (µg)	Total H_2_S Emission (µg)	NH_3_ + H_2_S Emission (µg)
Hyline	31.20 ^c^	2404.87 ^a^	551.43 ^a^	75.15 ^a^	17.30 ^a^	92.45 ^a^
Lohmann	25.88 ^d^	1720.54 ^c^	347.41 ^bc^	44.47 ^c^	8.92 ^b^	53.39 ^c^
Nongda	33.90 ^bc^	2032.32 ^b^	521.93 ^ab^	68.96 ^ab^	17.44 ^a^	86.40 ^b^
Jingfen	39.11 ^a^	1735.99 ^c^	492.63 ^bc^	67.86 ^b^	17.65 ^a^	85.42 ^b^
Xinghua	36.62 ^ab^	772.57 ^d^	318.41 ^c^	28.24 ^d^	11.70 ^b^	39.94 ^c^
Zhusi	36.49 ^ab^	1952.34 ^b^	528.88 ^ab^	71.24 ^ab^	19.09 ^a^	90.33 ^a^
SEM	0.02	30.60	56.81	2.18	1.71	2.57
*p*-value	<0.01	<0.01	<0.01	<0.01	<0.01	<0.01

^a–d^ Means of main effects without a common letter differ (*p* < 0.05).

**Table 2:** Body weights and productive performance of laying hens (*n* = 5).

Species	ADFI (g/day)	Egg Production (%)	Egg Weight (g)	FCR (g of Feed: g of Egg)
Hyline	112.87 ^b^	97.22 ^a^	53.53 ^a^	2.17 ^e^
Lohmann	120.13 ^a^	96.34 ^a^	53.52 ^a^	2.33 ^d^
Nongda	91.44 ^e^	83.96 ^c^	44.04 ^c^	2.47 ^c^
Jingfen	104.99 ^c^	91.89 ^b^	50.51 ^b^	2.26 ^d^
Xinghua	90.58 ^e^	75.34 ^d^	41.04 ^e^	2.93 ^b^
Zhusi	96.11 ^d^	70.06 ^e^	42.59 ^d^	3.22 ^a^
SEM	0.53	0.39	0.29	0.02
*p*-value	<0.01	<0.01	<0.01	<0.01

^a–e^ Means of main effects without a common letter differ (*p* < 0.05). ^1^ BW = Body weights; ADFI = Average daily feed intake; FCR = Feed conversion ratio.

**Table 3:** The volatile fatty acid (VFA) concentrations in six species of laying hens (*n* = 5).

Species	Acetic Acid (mmol/mL)	Propionic Acid (mmol/mL)	Isobutyric Acid (mmol/mL)	Butyrate Acid (mmol/mL)	Isovaleric Acid (mmol/mL)	Valeric Acid (mmol/mL)	Total VFAs (mmol/mL)
Hyline	49.12 ^a^	7.30 ^b^	0.71 ^b^	9.05 ^a^	1.96 ^b^	1.26 ^b^	69.47 ^a^
Lohmann	38.29 ^b^	5.87 ^d^	0.57 ^b^	7.52 ^b^	1.35 ^d^	0.91 ^c^	54.51 ^b^
Nongda	23.40 ^c^	3.61 ^e^	0.56 ^b^	5.49 ^c^	0.88 ^e^	0.56 ^d^	34.49 ^c^
Jingfen	41.48 ^b^	6.50 ^c^	0.65 ^b^	7.93 ^b^	1.70 ^c^	1.15 ^b^	59.41 ^b^
Xinghua	17.44 ^d^	2.69 ^f^	0.24 ^c^	4.98 ^c^	0.49 ^f^	0.35 ^e^	26.20 ^d^
Zhusi	52.13 ^a^	8.36 ^a^	1.39 ^a^	9.14 ^a^	2.46 ^a^	1.52 ^a^	75.00 ^a^
SEM	1.08	0.15	0.06	0.16	0.06	0.03	1.45
*p*-value	<0.01	<0.01	<0.01	<0.01	<0.01	<0.01	<0.01

^a–f^ Means of main effects without a common letter differ (*p* < 0.05).

**Table 4:** The biochemical caecal parameters of six species of laying hen (*n* = 5).

Species	pH	Urease Activity (mg/mL)	Ammonium Nitrogen (mg/mL)	Uric Acid (mmol/L)	Urea (mmol/L)
Hyline	7.72 ^bc^	0.20 ^a^	0.46 ^a^	0.31 ^a^	0.31 ^ab^
Lohmann	7.76 ^b^	0.27 ^a^	0.44 ^a^	0.35 ^a^	0.40 ^ab^
Nongda	7.65 ^c^	0.18 ^b^	0.46 ^a^	0.36 ^a^	0.44 ^a^
Jingfen	7.54 ^d^	0.12 ^b^	0.39 ^a^	0.17 ^b^	0.26 ^bc^
Xinghua	8.06 ^a^	0.15 ^b^	0.28 ^b^	0.12 ^b^	0.16 ^c^
Zhusi	7.64 ^cd^	0.13 ^b^	0.46 ^a^	0.16 ^b^	0.34 ^ab^
SEM	0.03	0.02	0.03	0.02	0.04
*p*-value	<0.01	0.01	0.01	<0.01	<0.01

^a–d^ Means of main effects without a common letter differ (*p* < 0.05).

**Table 5:** Spearman’s coefficient of correlation between odour emission and related caecum activities.

Gas Production	pH	Urease Activity (mg/mL)	Ammonium Nitrogen (mg/mL)	Uric Acid (mmol/L)	Number of ureC Genes	Reduction of SO_4_^2−^	Number of aprA Genes
Total volume (mL)	−0.43	−0.94 **	−0.37	−0.94 **	−0.77 ^†^	0.001	0.20
Concentration of NH_3_ (µg/L)	−0.33	0.03	0.93 **	0.67	0.47	N/A	N/A
Total NH_3_ emission (µg)	−0.89 *	−0.77 ^†^	0.90 **	0.47	−0.33	N/A	N/A
Concentration of H_2_S (µg/L)	−0.48	N/A	N/A	N/A	N/A	0.81 *	−0.46
Total H_2_S emission (µg)	−0.48	N/A	N/A	N/A	N/A	0.49	−0.35

^†^*p* < 0.10; * *p* < 0.05; ** *p* < 0.01.

## Supplementary Materials

The following are available online at https://www.mdpi.com/2076-2615/10/11/2172/s1. Table S1: Composition and nutrient levels of the basal diets, Table S2: Spearman’s correlation coefficient between the production performance and odour emission.
