# Supplementary material for: Effects of Different Laying Hen Species on Odour Emissions
Source: Animals (Basel). 2020 Nov 21;10(11):2172. doi: 10.3390/ani10112172 (PMC7700304; doi:10.3390/ani10112172)
Supplement: Supplementary file 1 [file animals-10-02172-s001.pdf]

Supplementary Material 1: Table S1 and Table S2.

Table S1. Composition and nutrient levels of the basal diets.

**Ingredients (%)**		**Calculated nutrient levels**	
Corn	62.0	ME（MJ/KG）[2]	11.5
Soybean meal	24.5	CP（%）	16.7
Wheat bran	1.00	Available phosphorus（%）	4.00
Limestone	8.00	Ca（%）	12.0
Salt	0.30	Lysine（%）	0.72
Methionine	0.15	Methionine（%）	0.36
Premix[1]	4.00	Methionine + Cysteine（%）	0.65

[1]Premix provided per kilogram of diet: vitamin A, 12,000 IU; vitamin D3, 4,000 IU; vitamin E, 32 IU; vitamin B1, 1.0 mg; vitamin B2, 7.0mg; vitaminB6, 1 mg; choline chloride, 500 mg; calcium pantothenate, 10 mg; pyridoxine HCl, 4.8 mg; biotin, 0.1 mg; folic acid, 1mg; vitamin B12, 0.014 mg; niacin, 35,000 mg; Fe (from ferrous sulfate), 60 mg; Cu (from copper sulfate), 10 mg; Mn (from manganese sulfate), 60 mg; Zn (from zinc sulfate), 70 mg; I (from calcium iodate), 0.35 mg; Se (from sodium selenite),0.15 mg.
[2]ME = metabolizable energy.

Table S2. Spearman's correlation coefficient between the production performance and odour emission.

Items	BW	ADFI	FCR	EP	EW	TVF	TVL	CN	CS	TN	TS	pH
BW	1	1.000**	-0.675	0.771†	0.886*	0.486	-0.600	0.200	0.257	0.257	-0.257	0.086
ADFI	-	1	-0.657	0.771†	0.886*	0.486	-0.600	0.200	0.257	0.257	-0.257	-0.086
FCR	-	-	1	-0.943**	-0.886*	-0.143	0.257	-0.371	-0.257	-0.257	0.257	0.086
EP	-	-	-	1	0.943**	0.086	-0.543	0.314	0.200	0.200	-0.486	0.143
EW	-	-	-	-	1	0.371	-0.600	0.486	0.429	0.429	-0.257	-0.086
TVF	-	-	-	-	-	1	-0.086	0.543	0.771†	0.771†	0.600	-0.600
TVL	-	-	-	-	-	-	1	-0.314	-0.257	-0.257	0.543	-0.429
CN	-	-	-	-	-	-	-	1	0.943**	0.943**	0.429	-0.429
CS	-	-	-	-	-	-	-	-	1	1.000**	0.543	-0.486
TN	-	-	-	-	-	-	-	-	-	1	0.543	-0.886*
TS	-	-	-	-	-	-	-	-	-	-	1	-0.486
pH	-	-	-	-	-	-	-	-	-	-	-	1

†P < 0.10; *P < 0.05; **P < 0.01; ***P < 0.001.

BW=Body weight; ADFI=Average daily feed intake; EP=Egg production; EW=Egg weight; FCR=Feed conversion ratio, TVL=Total gas volume, CN=Concentration of NH_3; CS=Concentration of H_2S; TN=Total NH_3; TS= Total H_2S; TVF=Total concentration of VFAs.
